# Pectus excavatum, kyphoscoliosis associated with thoracolumbar spinal stenosis: a rare case report and literature review

**DOI:** 10.1186/s12893-022-01716-7

**Published:** 2022-07-11

**Authors:** Sheng Zhao, Xuhong Xue, Kai Li, Feng Miao

**Affiliations:** Department of Orthopedics, The Second Hospital of Shanxi Medical University, No. 382 Wuyi Road, Shanxi 030001 Taiyuan, People’s Republic of China

**Keywords:** Pectus excavatum, Kyphoscoliosis, Thoracolumbar spinal stenosis, Case report, Literature review

## Abstract

**Background:**

Pectus excavatum is the most common congenital chest wall defect. Thoracolumbar spinal stenosis and kyphoscoliosis was seen in patients with pectus excavatum. It can be caused by ossification of the ligamentum flavum, which is rare in patients with pectus excavatum.

**Case presentation:**

We reported a 26-year-old woman presented bilateral lower extremities weakness and numbness for two months, progressive worsening. She was diagnosed as thoracolumbar spinal stenosis with ossification of the ligamentum flavum, thoracolumbar kyphoscoliosis associated with pectus excavatum. The posterior instrumentation, decompression with laminectomy, and de-kyposis procedure with multilevel ponte osteotomy were performed. Her postoperative course was uneventful and followed up regularly. Good neurologic symptoms improvement and spinal alignment were achieved.

**Conclusions:**

Pectus excavatum, kyphoscoliosis associated with thoracolumbar spinal stenosis is rare, and thus her treatment options are very challengeable. Extensive laminectomy decompression and de-kyphosis procedures can achieve good improvement of neurologic impingement and spinal alignment.

## Background

Pectus excavatum(PE) is a common congenital chest wall defect and characterized by central depression of lower part of sternum. It is displaced posteriorly into the chest cavity, producing a funnel-shaped chest [1]. Consequently, the anteroposterior distance of the thoracic cage is diminished, resulting in possible cardiac compression. The malformation can be asymmetric or symmetric; asymmetry associated with rotation of the sternum is usually the more depressed side [2]. Depending on the severity of the deformity, as well as the extent of cardiac involvement, most patients are asymptomatic; some may present with dyspnea, fatigue, cardiac arrhythmias or cardiopulmonary compromised. Even though absence of physical symptoms, some patients could develop psychological problems due to a distorted and ill-looking body appearance and require medical consultation [3].

The estimated incidence of PE is 0.1–0.3% [4, 5]. Although PE can be detected at birth or in early childhood, most of the patients may not present until early adolescence. Several studies reported that 15–22% of PE cases were accompanied by spine deformity [6–8], and other reports have demonstrated the coexistence of pectus excavatum and scoliosis, especially in connective tissue disorders such as Ehlers-Danlos syndrome, Marfan syndrome, and Noonan syndrome [9]. However, pectus excavatum associated thoracolumbar spinal stenosis is not common.

As the thoracic spine is relatively stable, myelopathy caused by thoracic spinal stenosis is much less common than in the cervical and lumbar spine. It can be caused by ossification of the ligamentum flavum, ossification of the posterior longitudinal ligament, posterior osteophytes, and thoracic intervertebral disc herniation. Furthermore, a combination of these factors may be responsible. We report a rare case presented with pectus excavatum, thoracic myelopathy caused by ossification of the ligamentum flavum, posterior osteophytes and thoracolumbar kyphoscoliosis.

## Case presentation

A 26-year-old woman visited our hospital with bilateral lower extremities weakness and numbness for two months, progressive worsening. One month ago, she couldn’t stand or walk without assistance. There was no history of trauma to the thoracolumbar region, and no relevant past interventions. She had no impairment of cognitive function or mental retardation. There was no exposure of known teratogenic agents and drugs. Her parents were healthy and their marriage was non-consanguineous. She had two unaffected older brother, and no known family history of PE, scoliosis or other problems. The pregnancy was uncomplicated, labor was spontaneous at 38 weeks and birth weight was 3350 g.

On physical examination, muscle testing revealed weakness of bilateral psoas and quadriceps with grades of 2/5, 2/5 in the left tibialis anterior, 1/5 in the right tibialis anterior, and 1/5 in bilateral toe extensors. She had paresthesias under the bilateral T12 distribution, as well as bowel bladder dysfunction. Her bilateral knee and ankle-jerks were hyper-reflexia; and Babinski sign were positive on both sides. The thoracolumbar spine was rigid with segmental kyphosis and absent extension when the patient bent backward. In addition to, uneven shoulders, back bulge, and trunk shift to the right were occurred.

**Fig. 1:**
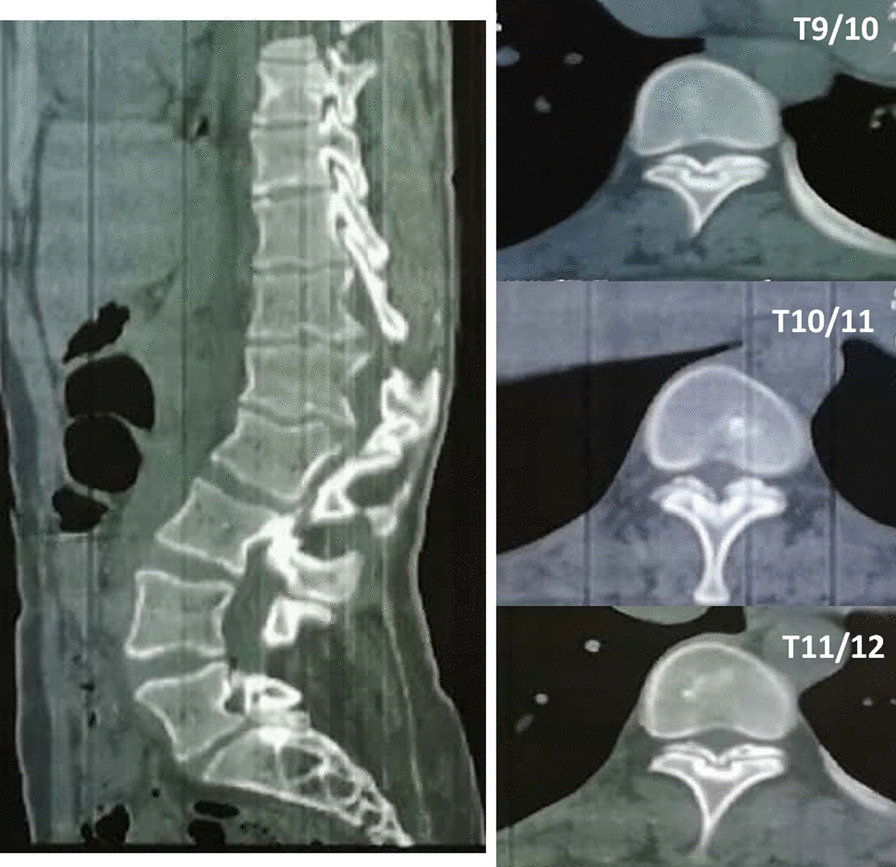
Sagittal and axial CT scans of the thoracic spine demonstrating thoracic spinal stenosis caused by multi-level ossification of ligamentum flavum and posterior osteophytes (T9/10, T10/11, T11/12)

**Fig. 2:**
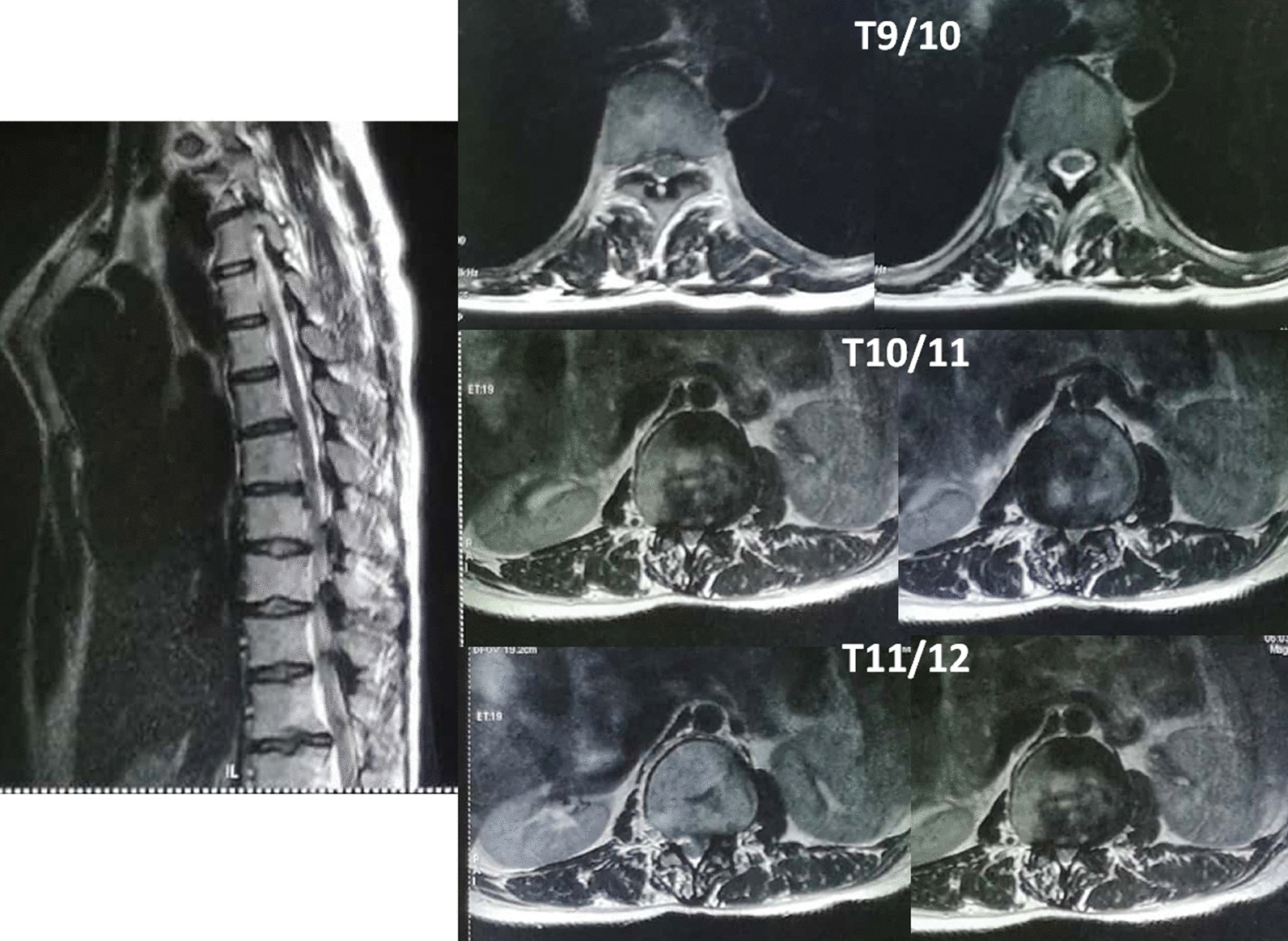
Sagittal and axial MRI of the thoracic spine showed multi-level severe thoracic spinal stenosis (T9/10, T10/11, T11/12) and spinal cord compression

**Fig. 3:**
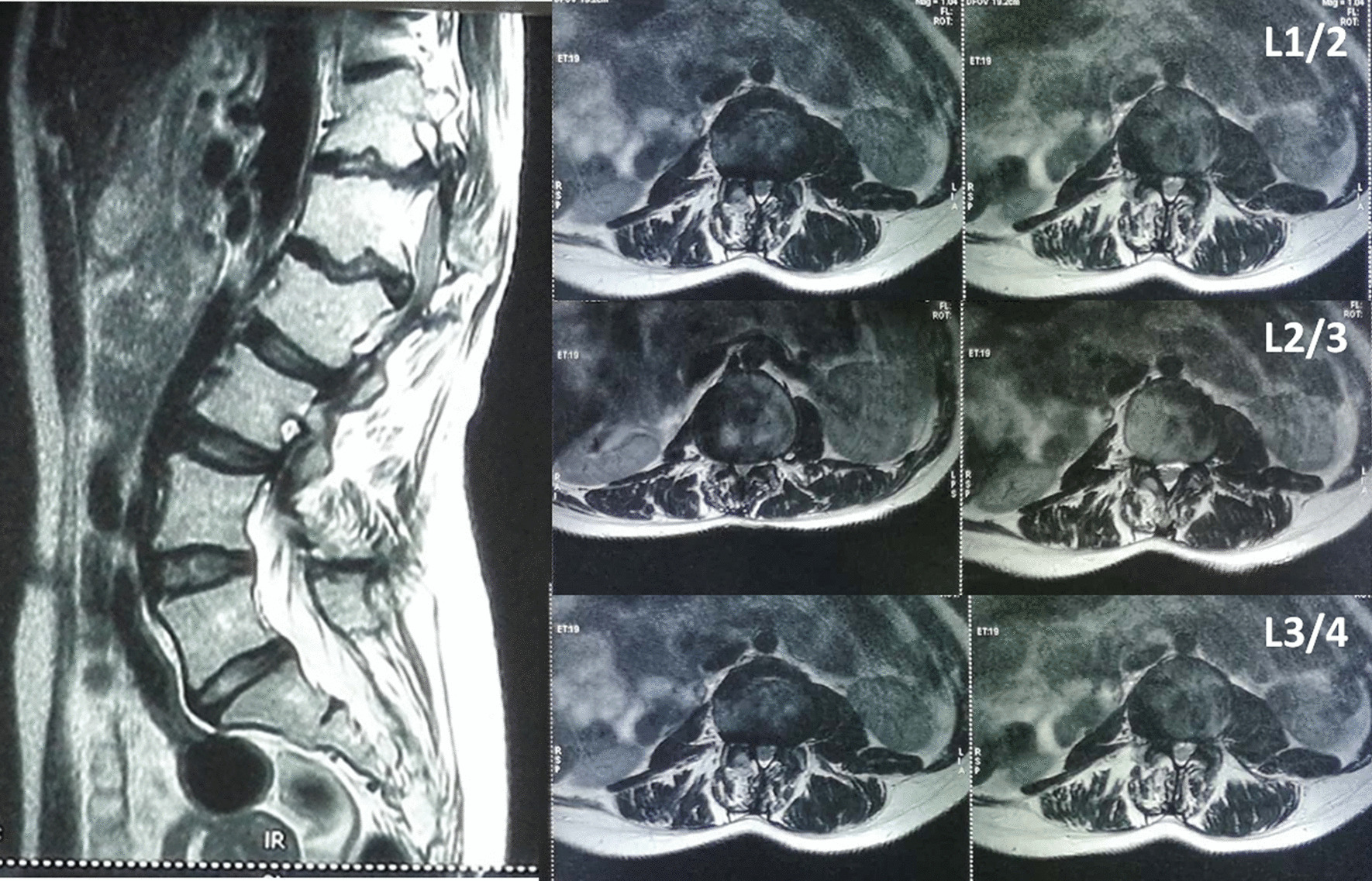
Sagittal and axial MRI of the lumbar spine showed multi-level severe upper lumbar spinal stenosis (L1/2,L2/3,L3/4) caused by posterior osteophytes and short pedicle

**Fig. 4:**
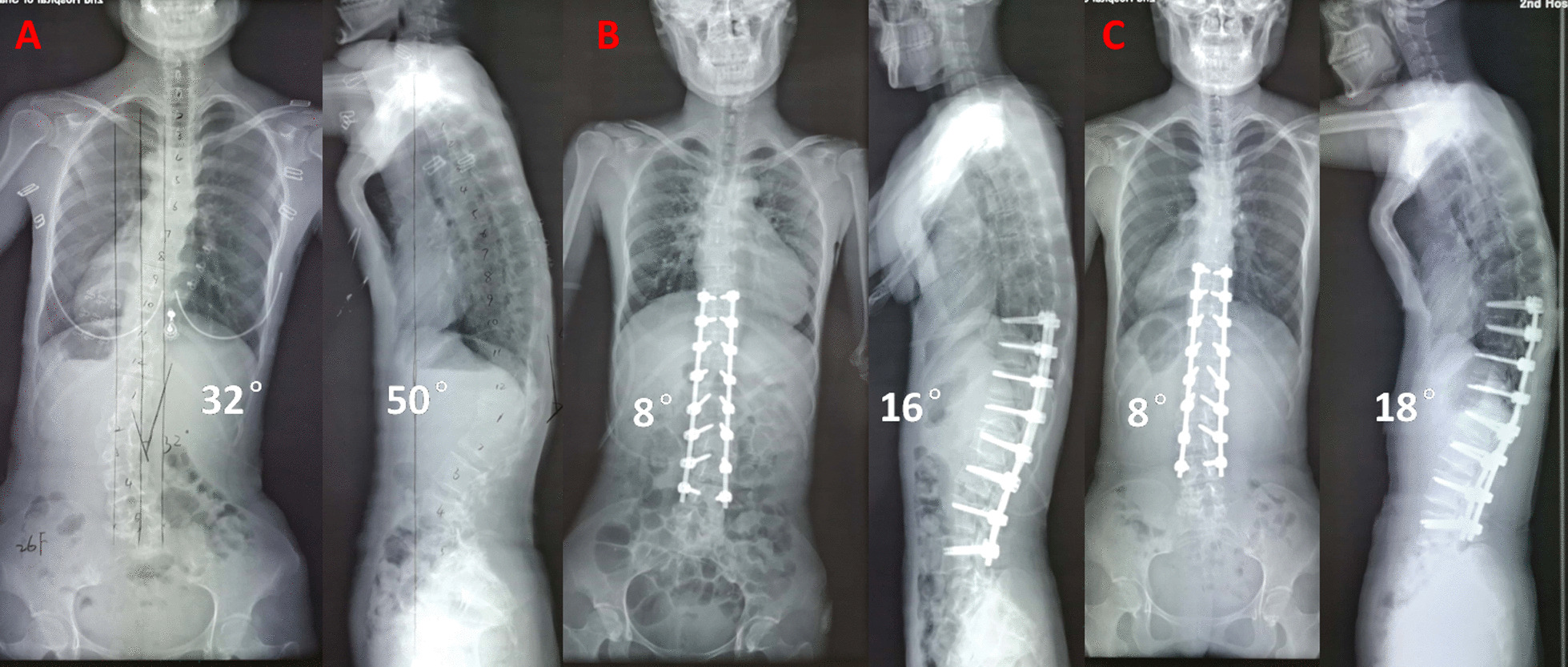
A 26-year-old female patient with thoracolumbar spinal stenosis, kyphoscoliosis and pectus excavatum. She underwent extensive laminectomy, ponte osteotomy and posterior fusion surgery (T9-L4). The kpphoscoliosis was improved significantly in preoperative and postoperative radiographs (**A,B**). At 6 months follow-up, the curve correction and spinal alignment were maintained very well(**C**)

**Fig. 5:**
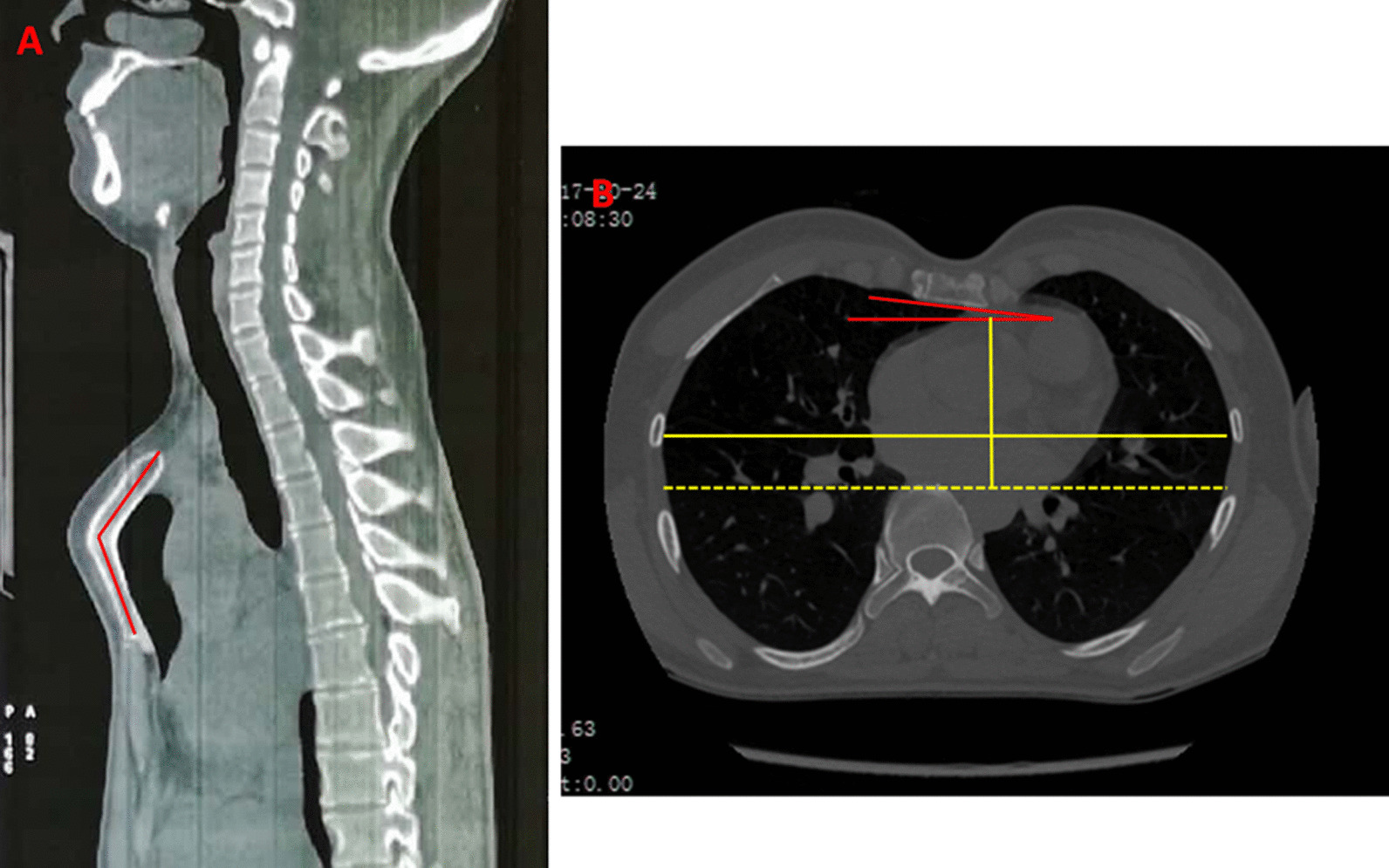
Sagittal CT scan showed the depressed sternum into thoracic cavity. The Louis angle (the angle between the manubrium and the body of the sternum) is 120 degree (**A**). The coronal CT scan demonstrated central depression of sternum with Haller Index of 3.4 and sternal rotation angle of 7 degree (**B**)

Neurological symptoms and signs suggested spinal cord compression. Thoracolumbar computed tomography scan showed multiple wedge-shaped vertebral bodies and posterior osteophytes from T12 to L2, lamina sclerosis and thickening in thoracic spine, ossification of ligamentum flavum at T9/10, T10/11 and T11/12, with a kyphotic curvature (Fig. 1). She had no history of lung or other disease. The routine laboratory test, pulmonary function test and echocardiography were normal. Magnetic resonance image of the spine showed multi-level spinal canal stenosis with cord compression at T10-L3 (Figs. 2 and 3). Her radiographs of the whole spine demonstrated thoracolumbar kyphoscoliosis from T10 to L2. The coronal scoliotic Cobb angle is 32° and segmental kyphotic Cobb angle is 50° (Fig. 4). Sagittal vertical axis (SVA) is positive with three centimeters forward the superior-posterior edge of S1 endplate (Fig. 4). In axial CT scan of lung, central depression of sternum produced a funnel-shaped chest. The anteroposterior distance of the thoracic cage is significant diminished. The Haller Index (width of the chest divided by the distance between the posterior surface of the sternum and the anterior surface of the spine [10] ) is 3.4. The sternum rotation is 7 degree on left side. The Louis angle (the angle between the manubrium and the body of the sternum [6]) is 120 degree (Fig. 5).

The surgery was suggested to rescue the function of spinal cord and correct the spinal deformity. A posterior multi-level laminectomy, ponte osteotomy (T11/12, T12/L1, L1/2), resection of the ossified ligamentum flavum, instrumentation, correction and bone graft fusion were performed from T9 to L4. The operation time was 270 min. The total amount of blood loss was 500 cc. During the operation, the signal of spinal cord monitoring was stable on baseline preoperatively. We found the MEP signals get better when spinal canal decompression is completed. The procedure went very well, no cardiopulmonary dysfunction or severe hypotension during her kyphoscoliosis correction and decompression surgery. Postoperatively, there was significant improvement in weakness of the bilateral lower extremities. Her postoperative course was uneventful and discharged in five days after surgery. The patient has been prescribed regular follow-up on 1, 3, 6, 12 months after surgery and every year further. The observation of neurological function was needed.

A postoperative X-ray demonstrated the lumbar scoliotic Cobb angle correction from 32° to 8°, the correction rate is 75%; and the focal kyphotic Cobb angle were from 50° to 16° in thoracolumbar region, the correction rate is 68% (Fig. 4). Neurologic examination of the lower extremities revealed a 5/5 muscle strength bilaterally and the patient was able to walk without assistance at 6 months after surgery. Well spinal balance in the sagittal and coronal planes was maintained (Fig. 4). Proximal kyphosis was increased in the upper thoracic spine from 18°to 24°when compared to the radiographs immediately postoperatively. We think the pose of upper extremities is one of the reasons when radiograph. Of course, further follow up was needed.

## Discussion and conclusions

Pectus excavatum was described as that the anterior chest wall is depressed into the thoracic cavity. It can manifest with asymptomatic, cosmetic issues or cardiopulmonary symptoms according on the extent of sternum malformation [11]. Although different hypotheses had been reported, the mechanism responsible for PE is still unclear. Studies have shown that patients with PE had shorter ribs on the more severely depressed side of the deformity. Therefore it may stem from unbalanced overgrowth in the costochondral regions [12, 13]. In addition, an intrinsic costochondral cartilage abnormality is possible due to the significant occurrence of pectus deformity in connective tissue disorders, such as Ehler-Danlos syndrome and Marfan syndrome [7]. It is worth mentioning that there is a genetic predisposition in patients with family history of pectus excavatum [14].

For the evaluation of PE, several parameters were reported in prior studies. Haller et al. suggested the ratio of transverse dimension of the chest to the sternovertebral distance in axial CT scan, which is named the Haller index or pectus index [10]. They suggested Haller index score is normal at 2.5 to 2.7 and severe at more than 3.25. A pectus index of 3.25 was predictive of need for surgical intervention. In fact, most patients with PE do not require surgery. However, Al-Qadi suggested that repair may be indicated in symptomatic patients with Haller index more than 3.5 and cardiopulmonary compromise. In our case, considering the Haller index is 3.4 without any cardiopulmonary symptom, surgical repair is not necessary. Another parameter is sternum torsion angle reported by Choi et al., which is the angle between the sternum and a horizontal line, with a positive value indicating counterclockwise rotation of the sternum and a negative value indicating clockwise rotation [15]. In our patient, the chest is basically symmetry and mild torsion; the sterna torsion angle is 7 degree.

Several studies have reported the incidence of scoliosis in association with PE. Waters et al. reported the incidence of scoliosis was 21.5% in 461 patients with PE [6]. Similarly, Wang et al. reported the incidence of scoliosis was 17.6% (25/142) with PE had scoliosis [8]. However, kyphosis or kyphoscoliosis in association with pectus excavatum was very rare. Only one study described the transversal tomography of funnel chest associated with kyphoscoliosis [16]. Ye et al. have used numerical stimulation technology to conduct to the process of minimally invasive correction in patient with PE and scoliosis. Their results have shown that correction of scoliosis and thoracic cavity abnormalities simultaneously could help to improve of the symptom of both PE and scoliosis [17]. However, there are risks of severe hypotension during scoliosis surgery. Because the corrective force applied and prone positioning intra-operatively may have a negative effect on cardiac function in these patients. Most patients with both scoliosis and PE have left side deviated sternum and a higher Haller index score, which can negatively impact cardiac function. Therefore, the hemodynamics and cardiac function of patients with scoliosis and PE should be monitored closely during scoliosis surgery [9].

Alexianu et al. reported a case with both scoliosis caused by neurofibromatosis and pectus excavatum. The patient became hypotensive in the prone position. When placed back in the supine position, her hypotension was improved. Subsequently posterior spinal fusion was performed successfully by using longitudinal bolsters to support the patient [18]. Another case of severe hypotension associated with prone positioning was reported by Bafus et al. in patient with scoliosis and PE [19]. A transesophageal echocardiogram performed by anesthesiologist revealed anterior compression of the right heart by the sternum. The patient again became hypotensive in prone position and had to returned to the supine position and the procedure was given up. After a modified Ravitch procedure to repair the PE, the patient was able to undergo posterior spinal fusion [20]. Therefore, they suggested that the fully assessment of the thoracic factors in patients with scoliosis and PE before surgery was of importance for orthopedic surgeons. On the contrary, in a study of Tauchi et al., none of patients experienced severe cardiopulmonary dysfunction or hypotension during their scoliosis correction surgery [9]. In our case, no significant hypotension and any emergency situations were found during spinal canal decompression and kyphoscoliosis corrective surgery.

Generally, thoracic spinal scoliosis caused by ossification of the ligamentum flavum, ossification of the posterior longitudinal ligament, posterior osteophytes, or thoracic intervertebral disc herniation were common in middle and old aged population. Even though for individuals with skeletal dysplasia, the thoracic mylopathy is not common at the early age. In patient with achondroplasia, congenital spinal stenosis usually was also found in adult. Previous studies had reported thoracolumbar kyphosis develops in up to 90% of patients with achondroplasia, which is usually associated with progressive neurologic impairment [21]. The features of our patient were focus on three points: firstly, mild skeletal dysplasia in vertebra body, skull and pelvic; secondly, thoracic mylopathy caused by ossification of the ligamentum flavum and posterior osteophytes; thirdly, thoracolumbar kyphoscoliosis was progressive, which contribute to spinal cannel stenosis. Even though it seems similar to achondroplasia in some ways, other signs and symptoms were not support the diagnosis of achondroplasia. According to the feature of images, multiple disc herniation, consecutive wedged vertebrae, irregular endplates and Schmorl’s nodes were showed in patient’s MRI. This patient meets atypical criteria of Scheuermann’s disease. However, thoracic and lumbar spinal stenosis caused by OLF and posterior osteophytes was not common in Scheuermann’s disease. Therefore, the accurate diagnosis for this case was still unknown. Gene test such as Next Generation Sequencing (NGS) may be helpful to premise diagnosis.

The aims of surgery were focus on two aspects for the patient with thoracic spinal stenosis, kyphoscoliosis and PE. One is spinal canal decompression in lower thoracic and lumbar spine; another is curvure and alignment correction in coronal and sagittal plane. For the surgery of thoracic spinal stenosis, there are usually high risk of neurological deteriorate. High rate of postoperative weakness and poor surgical outcomes have been reported after surgery with thoracic wide laminectomy only [22]. Another complication is dura tear. A congenital spinal canal stenosis, lamina thickens, and the resection of ossifying ligamentum flavum adhesion may increase the risk of this complication [23]. Fortunately, we performed a spinal decompression with a wide laminectomy using posterior instrumentation in this case and did not any complications.

To our knowledge, pectus excavatum, kyphoscoliosis associated with thoracolumbar spinal stenosis has not been documented previously. The treatment options are very challengeable because of its rarity. Extensive laminectomy, multi-level ponte osteotomy, and resection of the ossified ligamentum flavum were performed for decompression of neural impingement. Good neurologic symptoms improvement and spinal alignment were achieved with posterior instrumentation.

## Data Availability

The datasets used and/or analyzed during the current study are available from the corresponding author upon reasonable request.

## Abbreviations

PE: Pectus excavatum
SVA: Sagittal vertical axial
CT: Computed tomography
MRI: Magnetic resonance imaging
T: Thoracic
L: Lumbar
MEP: Motor-evoked potential
